# Harnessing hyperaccumulator plants to recover technology‐critical metals: where are we at?

**DOI:** 10.1111/nph.20449

**Published:** 2025-03-11

**Authors:** Elizabeth L. Rylott, Antony van der Ent

**Affiliations:** ^1^ Centre for Novel Agricultural Products, Department of Biology University of York York YO10 5DD UK; ^2^ Laboratory of Genetics Wageningen University and Research Wageningen 6708 PB the Netherlands; ^3^ Centre for Mined Land Rehabilitation, Sustainable Minerals Institute The University of Queensland Brisbane QLD 4072 Australia; ^4^ Laboratoire Sols et Environnement Université de Lorraine, INRAE, LSE Nancy F‐54000 France

**Keywords:** biomass, hyperaccumulator, metals, phytomining, phytoremediation, transporters

## Abstract

Since its inception over three decades ago, phytomining has finally reached the stage of commercial‐scale implementation, at least for nickel. Much potential remains to be realised for other elements, notably cobalt, selenium, and thallium, but this requires scientific impetus leveraging recent advances in insights garnered from molecular mechanisms of hyperaccumulation, domestication and agronomic development. These advances will also enable us to (genetically) improve hyperaccumulators for use in phytomining by targeted breeding, as well as synthetic biology approaches.

## Introduction

**Fig. 1:**
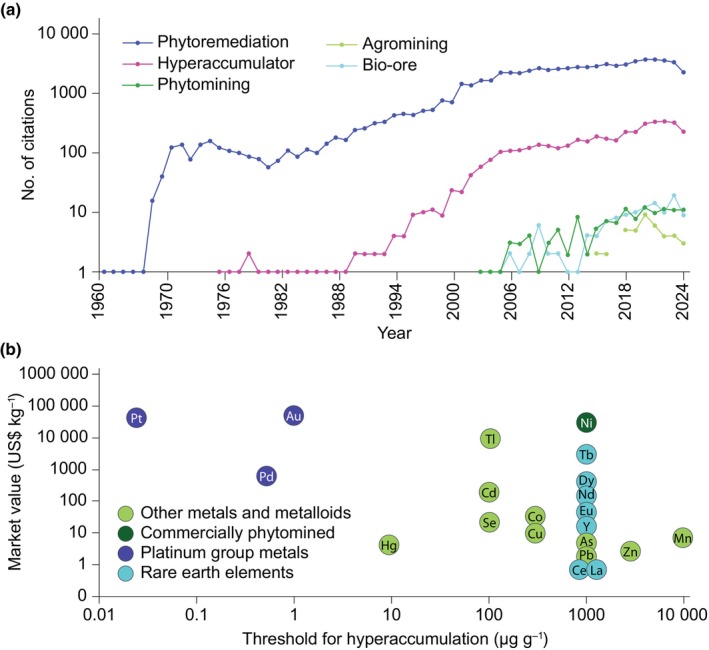
Emergence of phytomining and current market value of potential phytomining target elements. (a) Number of publications in National Center for Biotechnology Information search (http://dan.carlan.net/medline‐trend.html) for listed terms. Note that additional publications can be found in other online databases, and when using alternative term suffixes. (b) Market values for elements plotted against minimum defined threshold definition levels of hyperaccumulator species. Note that only exemplar rare earth elements (REEs) are shown and that both the individual or the sum of REEs make up the threshold value, see Supporting Information Table S1 for sources and further information.

Phytomining is the process of using plants to extract valuable metals and other elements from soils for financial gain (van der Ent *et al*., 2015a). As illustrated in Fig. 1(a), this technology has developed in the wake of phytoremediation: the use of plants to clean up contaminated environments by removing, detoxifying, or stabilising pollutants such as metals and organic compounds. First proposed by Rufus Chaney, a USDA agronomist, to phytoextract metals to remediate zinc (Zn) polluted land (Chaney *et al*., 1983), it was later expanded to phytomining as a concept to recover valuable metals, using gold (Au) as an exemplar (Anderson *et al*., 1998). Phytomining exploits natural hyperaccumulator plants, species that have evolved a remarkable ability to concentrate metals and metalloids in their living tissues to levels many times higher than in any other plant species growing on the same (geologically enriched) soils (Baker & Brooks, 1989). The widely accepted definition of trace element hyperaccumulators refers to plants that, when grown in their natural habitats (as opposed to metal‐amended or artificial media), contain elemental concentrations exceeding the defined threshold levels listed in Fig. 1(b) and Supporting Information Table S1 in their shoots and are capable of completing their lifecycle (van der Ent *et al*., 2013, 2015b).

### Environmental and remediation benefits

Besides economic extraction, phytomining offers the potential for significant environmental benefits by cleaning up soils and substrates contaminated by mining, industrial operations, and improper waste disposal. By recovering valuable metals, this technology has the potential to provide a financial return to cover the costs of site remediation (Rylott & Bruce, 2022). However, commercialisation of phytomining has been very slow to date and has undoubtedly been the source of substantial scepticism. While much research has made valuable contributions in this scientific area, an increasing number of studies have misinterpreted the definition of ‘hyperaccumulation’ and associated parameters and threaten to stall progress in the commercialisation of phytomining technology (van der Ent & Rylott, 2024).

### Economic viability and target elements

The economic viability of phytomining largely depends on: (1) existence of a suitable local hyperaccumulator species for the target element with high biomass, high metal accumulation, and agronomic adaptability; (2) the value of the target element produced by the phytomining operation; and (3) the presence of sufficient surface area of soil substantially enriched in the target element (van der Ent *et al*., 2015a). Taking these factors into consideration, only a few target elements and hyperaccumulator species can be envisaged to be phytomined commercially (Corzo Remigio *et al*., 2020), which are discussed below.

## Target elements

### Nickel, cobalt, thallium, and selenium

Fig. 1(b) plots metal uptake by hyperaccumulator species and metal prices. Research has primarily focussed on nickel (Ni), which is both relatively high value, and for which over 500 hyperaccumulating species in over 50 different families, have been identified, including the most well‐known Ni ‘metal crop’ species *Odontarrhena chalcidica* (formerly *Alyssum murale*) and also *Odontarrhena corsica* (formerly *Alyssum corsicum*) (Reeves *et al*., 2018). In common with many hyperaccumulator species, these plants exhibit relatively 'weedy' low biomass phenotypes and require significant breeding to improve agronomic traits (van der Ent *et al*., 2015a, b). Extensive field trials over a number of years have tested the Ni phytomining performance of *Odontarrhena* species, as well as *Bornmuellera emarginata*, in the field in Albania and Greece (Bani *et al*., 2015, 2021; Kyrkas *et al*., 2024); and more recently, trials have extended to the tropical regions in Malaysia using the ligneous shrub *Phyllanthus rufuschaneyi* (Bouman *et al*., 2018). Several start‐up companies have recently emerged that are now implementing Ni phytomining at the full commercial scale in Albania, Greece, and Malaysia using the aforementioned species. The recent impetus in commercial Ni phytomining is driven largely by the current Ni mining boom for electric vehicle  batteries resulting in a high Ni market price (*c*. $15 kg^−1^ in 2024). The very high carbon footprint of producing Ni, especially *via* pig iron in Indonesia (as much as 80–100 tonnes of carbon per tonne of Ni), and carbon credits under the EU Emissions Trading System for products that use Ni, it makes alternative low‐carbon footprint approaches, such as phytomining, attractive. Initially covered by a patent (granted in 1995 and then licensed), its expiry in 2015 meant that phytomining became an open technology (Chaney *et al*., 1995) and a workshop in 2015 at The University of Queensland culminating in a forward‐looking position paper on phytomining (van der Ent *et al*., 2015). More recently, the United States Department of Energy launched a funding programme on phytomining in 2024 which further spurred interest from investors and industry (ARPA‐E, 2024).

Often co‐located with Ni in geological occurrences such as in ultramafic soils, cobalt (Co) is increasing in value due to its use in the production of lithium‐ion batteries for electric vehicles; critical in the transition to a low‐carbon economy. Few genuine Co hyperaccumulator plants have been reported to date, the most significant is *Haumaniastrum robertii* from the Democratic Republic of the Congo, which may yield up to a calculated 25 kg Co per hectare yr^−1^ (Nkrumah *et al*., 2022); however, it has never been tested as a phytomining ‘metal crop’ and the only area in the world where it could be implemented at scale is in its native range in Central Africa where extensive natural Co‐enriched soils and Co‐contaminated mine wastes occur.

Although included in Fig. 1(b), there are no genuine reports for hyperaccumulation of Cr and Pb meeting all criteria including validation under controlled conditions (van der Ent *et al*., 2020). This is because both elements are highly unavailable for plant uptake in soils. However, there are numerous spurious studies where ‘hyperaccumulation’ is claimed based on soluble Cr(VI) or Pb(II) dosing in hydroponics (van der Ent *et al*., 2024).

Thallium (Tl) is a highly toxic metal with a significant economic value, and several hyperaccumulators have been documented including *Biscutella laevigata* (Reeves *et al*., 2018). Thallium phytomining could be a viable recovery method, and a way to remediate highly Tl‐contaminated soils, with (small‐scale) field studies yielding 16 kg Tl per hectare yr^−1^ (Anderson *et al*., 1999; Corzo Remigio *et al*., 2022). However, this will be a very niche market, given the global very limited demand for this metal and localised nature of Tl contaminated areas that are substantial in size (more than a few km^2^).

As with Ni, Co, and Tl, selenium (Se) hyperaccumulators have also been well‐documented (Reeves *et al*., 2018). *Astragalus bisulcatus* is able to yield a calculated 70 kg Se per hectare yr^−1^, and there is great potential in phytomining this element from the extensive natural seleniferous soils of Colorado/Wyoming in the United States for biofortification (Corzo Remigio *et al*., 2020). Again, no field‐scale testing of Se phytomining or domestication of *A. bisulcatus* or other Se hyperaccumulating species has happened to date.

### Rare earth elements

Rare earth elements (REEs) are a group of 16 naturally occuring metallic elements that share chemical similarities. This group is further subdivided into ‘light’ REEs: lanthanum (La) to europium (Eu), plus scandium (Sc); and ‘heavy’ REEs: gadolinium (Gd) to lutetium (Lu), plus yttrium (Y). The unusual geochemistry of the REEs means that these elements do not generally concentrate in deposits, except for carbonatite REE deposits and ion adsorption‐type REE deposits found, for example, in parts of China, Australia, and the United States. Elsewhere, REEs are spread at very dilute levels across the planet, and this dilution means that although these elements are actually not rare, they are difficult to mine and separate; processes that cause environmental damage and result in heavy carbon footprints.

The hyperaccumulation threshold for the REEs differs from all other elements, as it represents both the sum of REEs, as well as one of the individual REEs. However, the latter is unlikely to ever occur as the REEs in nature always co‐occur and plant accumulation is generally proportional to prevailing REE soil concentrations. Although a threshold of 1000 μg g^−1^ was originally suggested, it was lowered to 100 μg g^−1^ by van der Ent *et al*. (2021) in view of typical concentrations (< 10 μg g^−1^) in most plants. Many (sub)tropical pteridophytes notably *Dicranopteris linearis*, but also *Phytolacca americana* (pokeweed) and *Carya* spp. (hickory), can accumulate REEs above this threshold level (Dinh *et al*., 2022b). There may be many, as yet untested, plant species able to concentrate REEs even when growing in soils with background or relatively low REE concentrations. Studies have shown that plants can be used to accumulate REEs from mixed REE‐rich wastes such as coal fly ash (Dinh *et al*., 2022b) and soils (Liu *et al*., 2021). The REEs in particular have a market niche for separation technologies, for example the technology critical element terbium (Tb), currently worth over 2000‐fold more than cerium (Ce) (Fig. 1b; Table S1). There is some evidence of biological specificity, as studies in *P. americana* demonstrate that heavy REEs are preferentially translocated to leaves relative to light REEs (Grosjean *et al*., 2019), whereas the reverse has been observed in several fern species (Grosjean *et al*., 2020). Given increasing demand, geopolitical control, and the finite nature of REEs, it is possible that phytomining from REE‐wastes could make a small, but significant and environmentally greener, contribution to demand.

### Cadmium, mercury, and arsenic

Fig. 1(b) also lists several transition elements, notably cadmium (Cd) and the metalloid arsenic (As) for which hyperaccumulator species have been well‐documented (Reeves *et al.,* 2018) and mercury (Hg) for which there are no known genuine hyperaccumulators. Plants expressing bacterial genes encoding an organic Hg detoxification pathway were successfully demonstrated as a potential phytovolatilization route for the detoxification of concentrated Hg‐contaminated environments (Bizily *et al*., 2000). These relatively abundant elements have much lower market values, and when processing costs associated with the recovery of these elements are factored in, they become currently uneconomical to be phytomined and are thus not discussed further in this article. However, these relatively toxic elements are serious, global environmental pollutants, and with increasing public pressure and resultant changes in governmental regulations, environmental clean‐up in itself is becoming a valuable industry. The knowledge gained from developing phytomining research will continue to make active contributions to leverage development of financially viable phytoremediation technologies (Rylott & Bruce, 2022).

### Platinum group metals and gold

In comparison with Cd and As, the relatively high value of platinum group metals (PGMs) and Au should make them good candidates for phytomining; however, while they are high value, there are no documented natural hyperaccumulator species, and in Fig. 1(b), these elements cluster to the top left‐hand side of the graph. This absence of hyperaccumulators is not surprising given both the rarity of these elements in soils and that the chemistry of PGMs and Au means that they are unlikely to be present in biologically available, cationic forms in soils.

An often‐ignored issue to phytomining these precious metals is that approaches require the use of chemicals to induce metal solubility, allowing the oxidation of metal to cation forms that are biologically available for metal uptake. The most successful results have been obtained using cyanide, and PGM and Au tissue concentrations of *c*. 500 μg g^−1^ can be readily achieved (Dinh *et al*., 2022a). However, in mining industries, where this ‘cyanidation’ process is commonly used, poor management has resulted in huge environmental and health issues, particularly in developing countries (Cacciuttolo *et al*., 2023).

Centuries of mining activities have produced significant areas of metal‐contaminated tailings and other waste lands that now require restoration. These areas may contain untapped resources of technology‐critical metals that are uneconomical to extract using conventional methods (van der Ent *et al*., 2021; Nkrumah *et al*., 2022). While cyanidation techniques could never be done in the field (unless under controlled conditions on so‐called ‘leaching pads’), some plant and microbial species naturally produce cyanogenic compounds in the rhizosphere. If plants, ideally fast‐growing high‐biomass species, could be engineered to solubilise and accumulate PGMs and Au, phytomining could provide the necessary financial incentive to revegetate and restore these areas. Towards this goal, it is necessary to understand the complex interplay of physiological and biochemical processes regulating metal solubilisation and subsequent (hyper)accumulation.

## Hyperaccumulation mechanisms

There is much still to understand, including some key fundamental questions, on metal/metalloid hyperaccumulation. The current state of Ni and Se hyperaccumulation is well‐summarised by van der Pas & Ingle (2019) and Trippe 3^rd^ & Pilon‐Smits (2021), respectively, and summarised later. In any plant, accumulation of metal(loid)s above physiologically ‘normal’ levels causes oxidative stress with increased levels of reactive oxygen species. Transcriptomic studies on Se and Ni hyperaccumulators (Freeman *et al*., 2004; Meier *et al*., 2018; García de la Torre *et al*., 2021) show enhanced expression of genes encoding antioxidant activities, and increased levels of corresponding antioxidants such as glutathione, ascorbate, and flavonoids (Freeman *et al*., 2010; Wang *et al*., 2018; van der Pas & Ingle, 2019).

Hyperaccumulator plants have evolved specific adaptations that enable them to take up, translocate, and sequester exceptionally high levels of metal(loid)s. Kukier *et al.* (2004) and Risse *et al*. (2024) found that foliar Ni concentrations in hyperaccumulator species increased with increasing pH, an opposite finding to that reported in nonhyperaccumulators, which includes many crop species, where foliar Ni concentrations increase with decreasing soil pH. This is a predictable finding for crop plants; Ni is taken up in cation form, and as pH increases, this form is less available due to increased adsorption of Ni to soil phases and decreased H^+^ competition (van der Ent *et al*., 2016). Wenzel *et al*. (2003) suggested that the pH increase around the roots of *Noccaea goesingensis* could be from hydroxyl ions released during mineral dissolution of Mg‐ and Ni‐rich minerals; hydroxyl ions are known to be released during mineral weathering of ultramafic minerals (Chardot‐Jacques *et al*., 2013). In nature, tropical Ni hyperaccumulator species are known to preferentially inhabit soils with high available Ni with a relatively high soil pH (> 6.3), and strictly avoid acidic soils (van der Ent *et al*., 2016). There is evidence that hyperaccumulators recruit and support rhizosphere microbial communities that enhance uptake. Studies on *O. chalcidica* have shown that their rhizospheres host unique microbial populations, including Ni‐tolerant bacteria and plant growth‐promoting rhizobacteria (Durand *et al.,* 2023), with similar findings for the Se hyperaccumulator *Cardamine violifolia* (Yang *et al*., 2022). Furthermore, Se hyperaccumulators *S. pinnata* and *A. bisulcatus* actively seek out Se in the soil, a phenomenon described as ‘root foraging’ (Montanari *et al*., 2023).

In non‐hyperaccumulator species, metal from the soil into the root cell cytoplasm is driven by the broad substrate specificity ZIP (Zinc–Iron Permease) and NRAMPs (natural resistance‐associated macrophage protein) cell membrane transporters. These transporters take up predominantly not only zinc (Zn)–iron (Fe) and Mn, but also Ni, and their expression is enhanced in response to Ni. In the hyperaccumulator species *O. chalcidica*, *N. caerulescens* and *Senecio coronatus*, upregulation of the ZIP member IRT1 (Iron‐Regulated Transporter 1) enhances Ni uptake when compared to related species or accessions native to non‐Ni‐enriched soils (Meier *et al*., 2018; García de la Torre *et al*., 2021). An NRAMP:REE transporter NRAMP REE Transporter 1 (NREET1), from the REE hyperaccumulator fern *Dicranopteris linearis,* has recently been characterised and shown to compete with aluminium for uptake across the root plasma membrane (Zheng *et al*., 2023). Once in the root cytosol, members of the IRON‐REGULATED 1/FERROPORTIN family (IREG/FPN) have roles in the further transport in several Ni hyperaccumulator families (García de la Torre *et al*., 2021). IREG1 is primarily involved in the xylem loading of nickel, facilitating its long‐distance transport from roots to shoots, whereas IREG2 in the tonoplast membrane, functions to detoxify Ni *via* vacuolar sequestration in the shoots (Nishida, 2020). There is evidence that hyperaccumulators have optimised both transcript expression levels and specificity of metal transporters in the root cell membranes; for example, upregulation and duplication of IREG copies in Ni hyperaccumulator species relative to nonhyperaccumulators (García de la Torre *et al*., 2021). Furthermore, the Se hyperaccumulator *S. pinnata* contains one or possibly more, selenate‐specific transporters that are absent in related, but nonhyperaccumulator species (Harris *et al*., 2014). Transcriptomic studies indicate that several *S. pinnata* sulphate transporters (SULTRs), which also transport selenate, are constitutively upregulated while related nonhyperaccumulators regulate these transporters just in response to Se and S soil levels (Wang *et al*., 2018).

Plant metal tolerance proteins (MTPs) are tonoplast or cell membrane transporters that play a crucial role in maintaining metal ion homeostasis and are also implicated in metal hyperaccumulation. Expression of MTP1 from Zn hyperaccumulators *A. halleri* and *N. caerulescens* increases Zn shoot accumulation (Ricachenevsky *et al*., 2015). However, MTP1 is also upregulated in the Ni hyperaccumulator *N.* *goesingensis* (Salt, 2001). As Ni is transported as a free divalent cation, once inside the cell, it needs to be quickly bound to prevent damaging interactions with sensitive cellular components such as DNA proteins. Free metals are detoxified by chelation with ligands such as histidine, nicotianamine, and carboxylic acids such as citrate and malate. In nearly all Ni hyperaccumulator plants studied to date, Ni is complexed with citrate and/or malate (van der Pas & Ingle, 2019). However, as Ni is transported around the plant primarily as a free divalent cation in the xylem, it is not clear how the balance between chelation and free Ni ion is regulated (van der Pas & Ingle, 2019).

## Engineering improved, and novel, hyperaccumulators

**Fig. 2:**
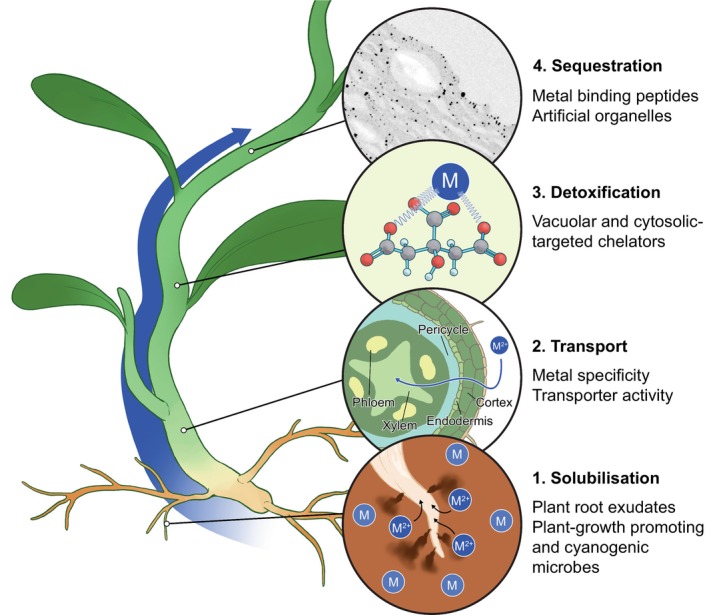
Schematic showing route of, and target points for engineering, metal (M) uptake, and storage in plants.

The rapidly evolving area of synthetic biology (‘synbio’) could offer the technological approach to engineering artificial hyperaccumulators, customised for metal choice, geographical location, and environmental conditions, and Fig. 2 highlights target areas for achieving this. Transporters described previously (IRT1, NREET1, IREG1 and 2, MTPs) are implicated in Ni transport as potential candidates on which to build synbio approaches. Machine‐learning algorithms present a paradigm shift in protein design and engineering. Tools such as RFdiffusion (Watson *et al*., 2023) and Chroma (Ingraham *et al*., 2023) have harnessed artificial intelligence (AI) to learn from thousands of protein structures in existing databases. These tools can then use algorithms such as ProteinMPNN to find a sequence that folds to the desired structure (Dauparas *et al*., 2022). RoseTTAFold (Baek *et al*., 2021) and, recently upgraded, AlphaFold3 (Abramson *et al*., 2024) can be used to optimise protein structure, and the resultant synthetic proteins can be expressed, purified, and tested.

Targets include metal selectivity, for example: by altering Ni transporters to favour Co; developing a Cd‐free Zn hyperaccumulating *N. caerulescens*, or uptake of elements not currently hyperaccumulated, such as Au and PGMs. The long‐term reach of synbio could be the production of artificial master regulators that control expression of a hyperaccumulator phenotype achieved by metal‐specific transporters, chelators, and antioxidant activities, with promoters directing expression at the required subcellular, tissue‐type, and organ levels. Such ambition requires long‐term public investment. Scientific bottlenecks include the need to understand the genetic and biochemical basis of Ni hyperaccumulation. Furthermore, much of the research to date has been conducted on the Brassicaceae, a family that contains well‐studied model (*Arabidopsis thaliana*) and crop (*Brassica napus*) species, and closely related hyperaccumulators (*N. caerulescens* and *O. chalcidica*). Extremely little is known about the biology of tropical high biomass shrub species, which have as‐yet unannotated genomes containing genome duplications and gene family expansions that make identifying orthologues of studied Brassicaceae genes challenging.

While agronomically improved Ni hyperaccumulators have been developed (van der Ent *et al*., 2015a), there is a lack of high biomass hyperaccumulator species that will grow, for example, in polluted environments in temperate regions. Towards this, transformation and regeneration methodologies need to be created, or further optimised for fast‐growing, high biomass (non‐hyperaccumulator) species such as *Salix* and *Populus* spp.

## Separation techniques for bio‐ore products

Once the phytomined biomass has been harvested, the metal‐rich biomass can be denoted as ‘bio‐ore’ and needs further processing to attain value. This is not often overlooked, but the bio‐ore has no value unless processed to a saleable product (e.g. pure Ni metal or highly pure Ni sulphate salt) and building a refinery suitable for processing bio‐ore requires a major capital investment. Currently, Ni is recovered from the hyperaccumulator biomass by ashing, with recovery of the associated heat for electricity generation, then either smelting to produce Ni metal alloys or acid leaching and purification to produce Ni sulphate. Ashing is the ‘least preferred’ approach and developing methods that do not release carbon back to the atmosphere are desirable. There are also potentially other ways to gain further value from the biomass. Nickel is a catalytically active metal, and studies show that when biologically bound within the plant tissue, its presence can be used to regulate the conversion of plant biomass to valuable platform chemicals (Chai *et al*., 2022) or enhance depolymerisation of plastics (Johar *et al*., 2023), with Ni recovered from the ash at the end of the process.

Chemistry‐based separation techniques for REEs are expensive and use environmentally damaging solvents and acids (Dinh *et al*., 2022b). An exciting new biological approach using lanmodulin‐binding proteins could make REE separation greener (Hussain *et al*., 2022), with the potential for synthetic biology approaches to further develop this approach to produce plants with REE uptake selectivity. For PGMs and Au, solubilisation with cyanogenic compounds is well‐known to increase uptake, with formation of metal nanoparticles in plant tissues. Accumulation of Au in *A. thaliana* has been demonstrated by overexpression of a Cu transporter, COPT2 (Tiwari *et al*., 2017). Beyond this, more recent research has expressed Au‐binding peptide sequences in *A. thaliana* to control the size of the resultant nanoparticles, with the aim of optimising catalytic activity in the subsequently pyrolyzed biomass (Loskarn *et al*., 2024). Palladium‐rich pyrolyzed biomass has activity as a catalyst for Suzuki–Miyaura reactions (cross‐coupling of a boronic acid to an organohalide; Chai *et al*., 2022).

In some hyperaccumulators, physical separation of metals occurs at the plant level, for example in the Ni hyperaccumulator *B. coddii*, Co localised in the basal leaves, whereas Ni localised to the top of the plant (Rue *et al*., 2020). This observation suggests that potential benefits for downstream recovery could also play an important role in breeding, or engineering, future hyperaccumulator plants. Together, these factors form the foundation of understanding phytomining processes, technologies, and their applications in both environmental remediation and resource recovery.

## What still needs to be done?

1. Investment is needed directly from the resource (mining) industries that own metal contaminated and/or naturally‐enriched sites, for example tailings dams or mineralised (ultramafic) soil around an operation. Such funding should be used to demonstrate commercial scalability with large‐scale field trials using locally native hyperaccumulator plant species to match the particular substrate and climatic conditions present at the site.
2. Start‐up companies are emerging with questionable practices attempting to leverage academic reputations to attract investment from nonexperts in speculative phytomining ventures. In reality, few academics possess the necessary expertise, experience, and local community relationships required to develop successful phytomining operations. Additionally, proposed methods, such as ‘artificially created’ hyperaccumulator plants, are far from commercialisation, and this strategy threatens the reputation of phytomining.
3. Public investment is also needed for fundamental research to understand the biology behind hyperaccumulation: deciphering the genetic basis, encoded biochemical activities and regulation; and for developing transformation and regeneration protocols for specific high biomass hyperaccumulator species.
4. Many hyperaccumulator species, and likely more as yet undiscovered, are unique to developing countries in the Global South where they are highly localised and rare, and often threatened by conventional mining activities. External investment and knowledge exchange is needed to support local academic institutes to build the necessary laboratory capacity and expertise to access their genetic resources compliant with the Nagoya Protocol for fair and equitable sharing of benefits.
5. Development of environmentally sustainable downstream techniques to valorise metal‐rich plant biomass into biofuels, platform chemicals, and recovered target metal.
6. At the global level a valid workable system to establish market definitions of metals derived from traditional mining and ‘Eco‐metal’ harvested from the emerging array of more environmentally sustainable alternatives, including phytomining. While phytomining will only ever contribute a minor percentage to the metal recovery industry, defined pricing structures of elements sourced in this way should confer a substantial, and increasing, premium. This premium would provide a strong impetus to develop meaningful collaborations between academics and industry to understand the barriers to rolling out this technology at scale. There already is precedent, with BMW paying above market rates for greener aluminium (Frangoul, 2021), Bellevue Gold working towards carbon neutral mining with the aim of selling ‘green’ gold (Dorrell, 2024), and in 2020, Elon Musk, chief executive of Tesla, promised a ‘giant contract for a long period of time’ to any company able to extract Ni in an efficient and environmentally sustainable manner (Sun & Burton, 2020).

There cannot be a greener mining process than using plants to ‘gently pluck’ metals from our environment, and metals produced this way would deliver the highest green premiums. With viable start‐up companies emerging, this exciting technology is now on the cusp of commercial implementation. However, further research and technological advancements are still necessary to address the current limitations and fully realise the potential of this innovative approach.

## Competing interests

None declared.

## Author contributions

ELR and AvdE contributed equally to the writing of this article.

## Disclaimer

The New Phytologist Foundation remains neutral with regard to jurisdictional claims in maps and in any institutional affiliations.

## Supporting information


**Table S1** Data on metal prices, threshold, and maximum recorded foliar values for named hyperaccumulator species, used to generate Fig. 1(b).
Please note: Wiley is not responsible for the content or functionality of any Supporting Information supplied by the authors. Any queries (other than missing material) should be directed to the *New Phytologist* Central Office.
