# Supplementary material for: Harnessing hyperaccumulator plants to recover technology‐critical metals: where are we at?
Source: New Phytol. 2025 Mar 11;246(3):859–66. doi: 10.1111/nph.20449 (PMC11982783; doi:10.1111/nph.20449)
Supplement: Supplementary file 1 — Table S1 Data on metal prices, threshold, and maximum recorded foliar values for named hyperaccumulator species, used to generate Fig. 1(b). Please note: Wiley is not responsible for the content or functionality of any Supporting Information supplied by the authors. Any queries (other than missing material) should be directed to the New Phytologist Central Office. [file NPH-246-859-s001.pdf]

New Phytologist Supporting Information
Article title: Harnessing hyperaccumulator plants to recover technology-critical metals: where are we at?
Authors: Elizabeth L. Rylott and Antony van der Ent
Article acceptance date: 2 January 2024

Element	Metal price (USGS 2023; US$/kg)	Threshold for hyperaccumulation in aerial tissues (µg/g[-1])	Reference for threshold value	Maximum recorded foliar value (µg/g[-1]) *summarised in Reeves *et al.* (2018);[#]van der Ent *et al*. (2020)[#]	Species with highest concentrations reported to date
As	4	1000	van der Ent et al. (2013)	23000*	*Pteris vittata*
Au	57878	1	Baker & Brooks (1989)	0.78[#]	*Pinus pinea*
Cd	143	100	van der Ent et al. (2013)	3600*	*Arabidopsis halleri*
Co	33	300	van der Ent et al. (2013)	10000*	*Haumaniastrum robertii*
Cu	9	300	van der Ent et al. (2013)	14000*	*Aeolanthus biformifolius*
Hg	33	10	Santos et al. (2010)	1276[#]	*Polypogon monspeliensis*
Mn	7	10000	Baker & Brooks (1989)	55000*	*Virotia neurophylla*
Ni	27558	1000	Brooks et al. (1977)	76000*	*Berkheya coddii*
Pb	2	1000	van der Ent et al. 2013	8000*	*Noccaea rotondifolia subsp. cepaeifolia*
Pd	707	-	Dinh et al 2022	0.71[#]	*Berkheya coddii*
Pt	31511	-	Dinh et al 2022	0.03[#]	*Taraxacum officinale*

Ce	1	1000	van der Ent et al. (2013)	7000*	*Dicranopteris linearis*
Dy	390				
Eu	30				
La	1				
Nd	130				
Tb	2000				
Y	13				
Se	21	100	van der Ent et al. 2013	15000*	*Astragalus bisulcatus*
Tl	9400	100	van der Ent et al. 2013	19000*	*Biscutella laevigata*
Zn	4	3000	van der Ent et al. 2013	54000*	*Noccaea caerulescens*

Table S1. Data on metal prices, threshold, and maximum recorded foliar values for named hyperaccumulator species, used to generate Figure 1b. Summarised in [*]Reeves *et al.* (2018);[#]van der Ent *et al.* (2020), [§]mean total REEs. USGS, U.S. Geological Survey.

References

Baker AJM, Brooks RR. 1989. Terrestrial higher plants which hyperaccumulate metallic elements - A review of their distribution, ecology and phytochemistry. *Biorecovery* **1**: 81-126.

Brooks RR, Lee J, Reeves RD, Jaffre T. 1977. Detection of nickeliferous rocks by analysis of herbarium specimens of indicator plants. *Journal of Geochemical Exploration* 7: 49–57.

Dinh T, Dobo Z, Kovacs H. 2022. Phytomining of noble metals – A review. *Chemosphere* **286**: 131805.

Reeves RD, Baker AJM, Jaffré T, Erskine PD, Echevarria G, van der Ent A. 2018. A global database for plants that hyperaccumulate metal and metalloid trace elements. *New Phytologist* **218**: 407-411.

Santos GCGd, Rodella AA, Abreu CAd, Coscione AR. 2010. Vegetable species for phytoextraction of boron, copper, lead, manganese and zinc from contaminated soil. *Scientia Agricola* 67.

U.S. Geological Survey, 2023, Mineral commodity summaries 2023: *U.S. Geological Survey*, 210 pp., https://doi.org/10.3133/mcs2023.

van der Ent A, Baker AJM, Reeves RD, Pollard AJ, Schat H. 2013. Hyperaccumulators of metal and metalloid trace elements: Facts and fiction. *Plant and Soil* **362**: 319-334.

van der Ent A, Pollard AJ, Echevarria G, Abubakari F, Erskine PD, Baker AJM, Reeves RD. 2020. Exceptional Uptake and Accumulation of Chemical Elements in Plants: Extending the Hyperaccumulation Paradigm. In: “Agromining: Farming for Metals” Mineral Resource Reviews series, (second edition) van der Ent A, Baker AJM, Echevarria G, Simonnot M-O, Morel JL (Eds.), Springer International Publishing, pp 99–131.
